# Letter to the Editor in response to “Course and predictors of posttraumatic stress-related symptoms among family members of deceased ICU patients during the first year of bereavement”

**DOI:** 10.1186/s13054-021-03806-z

**Published:** 2021-11-10

**Authors:** Susumu Yagome, Michihiro Tsubaki, Yoshiyasu Ito

**Affiliations:** 1 Department of Health Data Science, Yokohama City University Graduate School of Data Science, 2-2-1 Minatomirai, Nishi-ku, Yokohama, 220-8107 Japan; 2 School of Nursing, Kitasato University, 1-15-1 Kitasato, Minami-ku, Sagamihara City, Kanagawa 252-0373 Japan; 3 College of Nursing Art and Science, University of Hyogo, 3-71 Kitaoujicho, Akashi City, Hyogo 673-0021 Japan

**Dear Editor,**

We read with great interest of the report by Tang et al. [1] about the course and predictors of clinically significant PTSD symptoms among family members of deceased ICU patients. As for the multiple predictors reported, the results were very important in predicting PTSD in families experiencing bereavement in the ICU and indicating modifiable end-of-life care to prevent it. However, from our point of view, the method of selecting and evaluating the independent variables warrants further study.

Usually, when identifying predictors, it is necessary to evaluate interactions and multicollinearity within the independent variables and to improve the performance of the modeling through a process of variable selection such as stepwise. In this study, although the details of the variables selected by the authors are explained in detail, the relationships among the variables and the selection criteria are not discussed. Evaluating variables improves not only the performance of modeling but also has the advantage of reducing the number of variables to be captured and applied in practice. The author will need to discuss the discussion on variable evaluation. This study could be used to model the prediction of PTSD in families who have experienced bereavement in the ICU. To do so, it is also necessary to discuss the external validity of the obtained model. In this study, only the construction of the model was conducted, and external validity was not examined. It is necessary to discuss whether or not to examine the external validity of the model in the same characteristic group, as has been partially discussed in the literature review. PICS-F, including PTSD in families who have experienced bereavement in the ICU, is becoming more well known but not well proven [2]. A more advanced model of PTSD prediction for families who have experienced bereavement in the ICU is expected to overcome this challenge.

## Data Availability

Not applicable.
