# Supplementary material for: Letter to the Editor in response to “Course and predictors of posttraumatic stress-related symptoms among family members of deceased ICU patients during the first year of bereavement”
Source: Crit Care. 2021 Nov 10;25:384. doi: 10.1186/s13054-021-03806-z (PMC8579567; doi:10.1186/s13054-021-03806-z)
Supplement: Related texts

# Reply: Authors’ response

**Authors:** Siew Tzuh Tang, Fur-Hsing Wen

**Affiliations:** 4 grid.145695.a 0000 0004 1798 0922 School of Nursing, Chang Gung University, Taoyuan City, Taiwan; 5 grid.445078.a 0000 0001 2290 4690 Department of International Business, Soochow University, Suzhou, Taiwan

We appreciate the interest expressed and comments provided by Dr. Yagome and colleagues to our publication. A more advanced model of prediction of severe PTSD symptoms for bereaved family members of ICU decedents was suggested by examining interactions and multicollinearity among the proposed independent variables, selecting variables through stepwise regression, and evaluating external validity of our final model.

We aimed to exploratorily and comprehensively examine the predictors of severe PTSD symptoms for bereaved family members of ICU decedents from immutable family and patient characteristics and modifiable factors of EOL care in ICUs to fill the gap of current limited and inconclusive knowledge about factors associated with ICU bereaved family members’ severe PTSD symptoms as indicated in the Introduction section. Without a well-established theory [3] or conceptual model guided by the existing studies for factors associated with ICU bereaved family members’ severe PTSD symptoms, interactions among variables to identify moderation effects of specific variables (which increase the chance of multicollinearity [4]—a concern also raised in the letter) were not examined in our model. By the same token, instead of stepwise regression, we used simultaneous regression model—an automatic procedure for statistical model selection when there is a large number of potential explanatory variables and no underlying logical or theoretical basis on which to set the criteria for the variable selection [5]. We recognize that there may be potential multicollinearity among our proposed variables, but we did not address this issue by preliminarily omitting some variables that may be highly correlated with other variables to avoid running a type II error for our explanatory study. We recognize the necessity of further external validation of our research findings to enhance generalizability of our results to national population who share basic characteristics of our sample and ICU bereaved family members from countries where cultural, societal, and healthcare characteristics are substantially different from Taiwan, especially considering cultural variations in grief reactions during bereavement in Western and Eastern countries.
